# Automatic diagnosis of neurological diseases using MEG signals with a deep neural network

**DOI:** 10.1038/s41598-019-41500-x

**Published:** 2019-03-25

**Authors:** Jo Aoe, Ryohei Fukuma, Takufumi Yanagisawa, Tatsuya Harada, Masataka Tanaka, Maki Kobayashi, You Inoue, Shota Yamamoto, Yuichiro Ohnishi, Haruhiko Kishima

**Affiliations:** 1 Osaka University Institute for Advanced Co-Creation Studies, Suita, Japan; 2 Department of Neurosurgery, Osaka University Graduate School of Medicine, Suita, Japan; 3 JST PRESTO, Suita, Japan; 4 Department of Mechano-Informatics, Graduate School of Information Science and Technology, The University of Tokyo, Tokyo, Japan; 5 RIKEN, Tokyo, Japan

## Abstract

The application of deep learning to neuroimaging big data will help develop computer-aided diagnosis of neurological diseases. Pattern recognition using deep learning can extract features of neuroimaging signals unique to various neurological diseases, leading to better diagnoses. In this study, we developed MNet, a novel deep neural network to classify multiple neurological diseases using resting-state magnetoencephalography (MEG) signals. We used the MEG signals of 67 healthy subjects, 26 patients with spinal cord injury, and 140 patients with epilepsy to train and test the network using 10-fold cross-validation. The trained MNet succeeded in classifying the healthy subjects and those with the two neurological diseases with an accuracy of 70.7 ± 10.6%, which significantly exceeded the accuracy of 63.4 ± 12.7% calculated from relative powers of six frequency bands (δ: 1–4 Hz; θ: 4–8 Hz; low-α: 8–10 Hz; high-α: 10–13 Hz; β: 13–30 Hz; low-γ: 30–50 Hz) for each channel using a support vector machine as a classifier (*p* = 4.2 × 10^−2^). The specificity of classification for each disease ranged from 86–94%. Our results suggest that this technique would be useful for developing a classifier that will improve neurological diagnoses and allow high specificity in identifying diseases.

## Introduction

Computer-aided diagnosis is crucial to improve treatment strategies for neurological diseases^1,2^. Various systems have been developed to classify healthy subjects and patients with diseases such as epilepsy^3,4^, Alzheimer’s disease^5,6^, Parkinson’s disease^7,8^, multiple sclerosis^9,10^, autism spectrum disorders^11,12^, brain tumours^13,14^, alcoholism related disorders^15,16^, and sleep disorders^17,18^. Recent advances in pattern recognition using the deep learning method^19^ enable the classification of various imaging data, such as magnetic resonance imaging (MRI) of Alzheimer’s disease^20^ and brain tumours^21^, lung cancer X-rays^22^, and patient symptoms^23,24^. We expect deep learning can extract the features unique to various neurological diseases from the data^19^ and surpass human ability to classify that data^25^. This system will improve the treatment of neurological diseases by reducing the doctor’s burden and increasing the accuracy of diagnosis using a large volume and high dimension of neuroimaging data, which sometimes make diagnosis difficult, inefficient, and, even worse, can cause human errors^1,26,27^.

Magnetoencephalography (MEG) and electroencephalography (EEG) are essential to the diagnosis of epilepsy^28^ and useful in characterizing various neurological diseases such as Parkinson’s disease^29^ and Alzheimer’s disease. MEG has a higher signal-to-noise ratio^30^ and a higher spatial resolution^31^ than EEG, which allows precise monitoring of cortical activity^32^. However, diagnosis using MEG is often burdensome for doctors and requires some experience due to the large number of sensors, complicated pre-processing necessary to extract cortical signals, and the difficulty in classifying various waveform patterns. The classification of MEG signals using deep learning will reduce the burden on doctors and improve the accuracy of neurological diagnoses.

**Figure 1:**
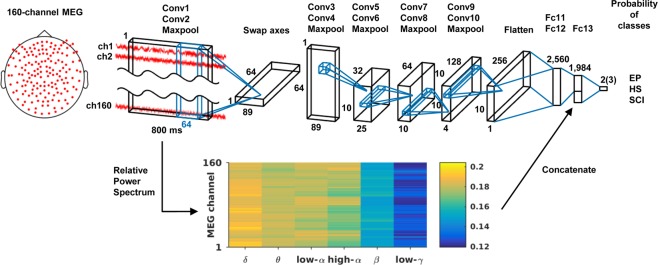
Brief architecture of the MNet. Features extracted by the convolutional layers and the relative powers of the six frequency bands are concatenated before fully connected layer 13. Output size depends on classification patterns: two for binary classification and three for classification of two diseases and healthy subjects. Conv: convolutional layer; Fc: fully connected layer; HS: healthy subjects; EP: patients with epilepsy; SCI: patients with spinal cord injury.

In this study, we have developed MNet, a novel deep neural network to classify multiple neurological diseases using resting-state MEG signals (Fig. 1). MNet is designed to extract global features of 160 channels of raw MEG signals by applying a large kernel over 64-ms whole channels at the first convolution layer, where the following layers were designed to extract time-frequency components of the global features. In addition, some band powers of 800-ms MEG signals were used as an input to the fully connected layer as these are classic features known to be informative for the classification of diseases^8,33^. We used the MEG signals of 67 healthy subjects (35 women and 32 men, median age 60 years, range 21–86 years), 26 patients with spinal cord injury (SCI; 3 women and 23 men, median age 34.5 years, range 22–61 years), and 140 patients with epilepsy (72 women and 68 men, median age 26.5 years, range 7–71 years) to train and test the network using cross-validation. We selected epilepsy as a benchmark because many previous studies have demonstrated the identification of epilepsy using EEG^34^; to compare classification accuracy to these EEG studies, we only used interictal MEG signals for patients with epilepsy. We included SCI as a neurologic disorder without brain damage^35^. We evaluated the MNet’s classification accuracy for these subjects, and for comparison, we also classified them using a support vector machine (SVM) with the same band powers used in the fully connected layer of the MNet. We hypothesized that the MNet exceeds the SVM in classification accuracy through use of the global features from the raw signals.

## Results

### Classification of multiple neurological diseases

The MNet was trained with resting-state MEG signals to classify healthy subjects, patients with epilepsy, and patients with SCI using 10-fold cross-validation and hyperparameters selected based on our preliminary experiments on a different dataset. The classification accuracy for labelling the three different types of subjects was 70.7 ± 10.6% (mean ± SD; accuracy of each fold: 88.9%, 84.1%, 61.1%, 73.8%, 57.1%, 63.5%, 73.8%, 73.0%, 73.0%, and 58.7%). The three different subject labels were also classified using only the relative powers of six frequency bands for each channel by SVM. The classification accuracy using only the relative powers was 63.4 ± 12.7% (accuracy of each fold: 65.9%, 77.0%, 49.2%, 65.1%, 59.5%, 68.3%, 54.8%, 89.7%, 54.0%, and 50.8%), which is significantly lower than that using the MNet (*p* = 4.2 × 10^–2^, single-sided Wilcoxon signed-rank test^36^). The sensitivity and specificity for each disease are shown in Table 1 (see Supplementary Tables S1 for MNet confusion matrix). The MNet classified two neurological diseases with a specificity exceeding 86.0%. Notably, the MNet classified epilepsy patients with a sensitivity of 87.9%. These results demonstrate that the MNet is useful for the specification of various neurological diseases using MEG signals, as well as for detecting epilepsy patients.

**Table 1:** Sensitivity and specificity to classify three disease labels of subjects using MNet.

	Sensitivity (%)	Specificity (%)
EP	87.9	86.0
HS	79.1	88.0
SCI	46.2	94.2

HS: Healthy subjects; SCI: Patients with spinal cord injury; EP: Patients with epilepsy.

**Figure 2:**
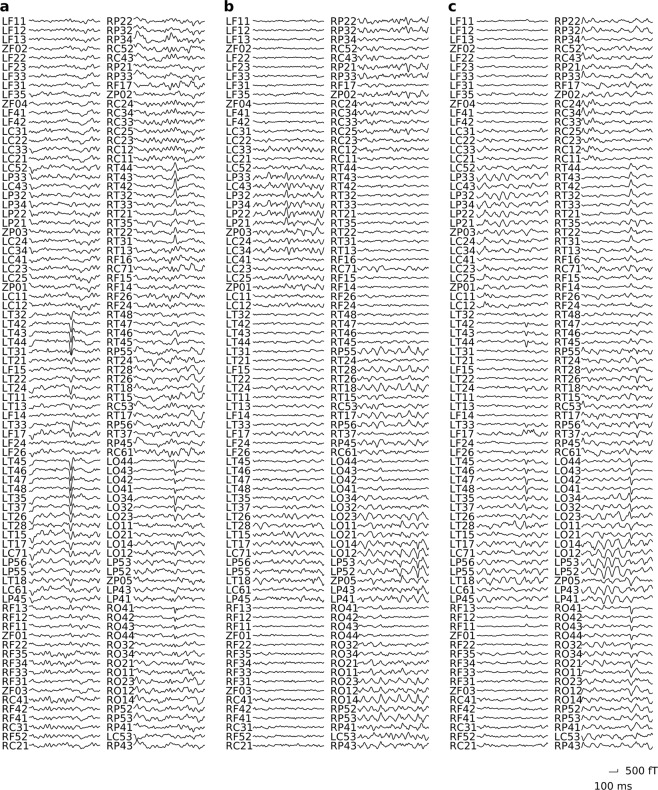
MEG signals labelled with high probability by MNet. The figure shows representative 800-ms MEG signals that were correctly classified by the MNet with high probability for a (**a**) patient with epilepsy, (**b**) healthy subject, and (**c**) patient with spinal cord injury. The probabilities of their labels were 99.9%, 99.1%, and 83.2%, respectively. The descriptions located at the left of waves (LF11 to RP43) indicate the MEG channel positions.

**Figure 3:**
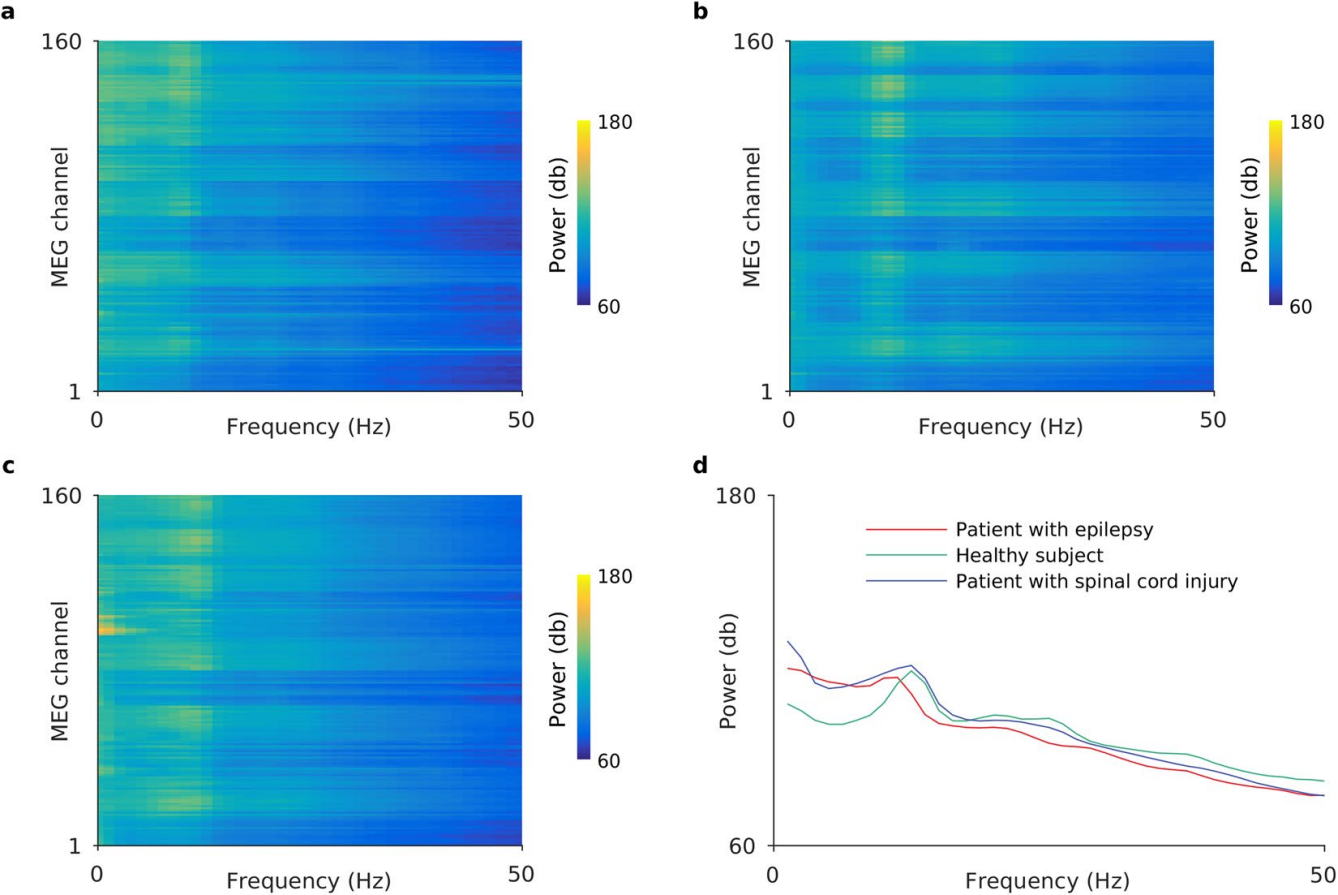
Power spectrums of MEG signals labelled with high probability by MNet. Panels (a–c) show the log power spectrums of the whole MEG signals of the same subjects as Fig. 2. Color represents the logarithm of power; (**d**) shows the log power spectrum averaged over all channels shown in (**a**–**c**). In all cases, the logarithm of power was calculated by applying Welch’s power spectral density estimate using a Hamming window of length 800 ms for each channel, and by taking logarithms.

Representative MEG signals that were correctly classified by the MNet for each disease are shown in Figs 2 and 3. Each panel shows an example of an 800-ms segment of raw MEG signals and the log power spectrums from a subject that was correctly classified with high probability among each subject group from the different disease labels. There were no spikes or particular abnormal waveforms in these examples. It was suggested that the MNet successfully classified the MEG signals using features that were not used in the usual diagnosis based on waveforms.

In the classification of diseases, we used the fixed hyperparameter (weight decay: 0.0005) determined from our previous research. To evaluate how the hyperparameter of weight decay affects classification accuracy, we compared the accuracies using three different weight decay parameters: 0.005, 0.0005, and 0.00005. As shown in Table 2, the mean of the squared errors was lowest with the weight decay of 0.0005, which shows that our hyperparameter choice was reasonable.

**Table 2:** Errors of 10-fold cross-validation by weight decay.

Weight decay	Mean(Error)	SD(Error)	Mean(Error^2^)
0.005	34.1%	12.9%	13.3%^2^
0.0005	29.3%	10.6%	9.7%^2^
0.00005	31.3%	14.2%	11.8%^2^

Mean(Error): average of error over 10-fold cross-validation; SD(Error): standard deviation of error over 10-fold cross-validation; Mean(Error^2^): average of squared error over 10-fold cross-validation.

### Classification of patients with each disease and healthy subjects

To compare the classification accuracy of the MNet with the accuracy of previously reported systems, the MNet was trained for binary classification of two disease labels for healthy subjects, patients with epilepsy, and SCI. The classification accuracies by the MNet and the accuracies for the same combination of subjects using SVM are shown in Table 3 (see Supplementary Tables S2, S3 and S4 for confusion matrices for MNet classifications; see also Supplementary Figs S6, S7 and S8 for the respective ROC curves). The MNet classified healthy subjects and patients with epilepsy with significantly higher accuracy than the SVM with the relative powers for each channel (*p* = 4.0 × 10^−2^, single-sided Wilcoxon signed-rank test).

**Table 3:** Binary classification accuracies using the MNet and SVM.

	MNet Accuracy (%)	SVM Accuracy (%)	*p*-value
HS vs. EP	88.7 ± 9.3	83.6 ± 7.8	4.2 × 10^−2^
HS vs. SCI	60.4 ± 16.1	61.4 ± 17.4	5.7 × 10^−1^
EP vs. SCI	79.8 ± 11.7	77.4 ± 13.4	9.3 × 10^−2^

HS: Healthy subjects; SCI: Patients with spinal cord injury; EP: Patients with epilepsy.

### Classification of patients with epilepsy and healthy subjects by nested cross-validation

To examine whether the hyperparameters used in the classification were optimal, we performed three-fold nested cross-validation to classify patients with epilepsy and healthy subjects by optimizing the hyperparameters weight decay and epoch in each inner loop. For each of the three folds, the hyperparameters were selected as follows: weight decay, 0.0005, 0.00005, and 0.0005; epochs, 15, 19, and 26. The selected hyperparameters were similar to those used in the 10-fold cross-validation with fixed hyperparameters (weight decay, 0.0005; epochs, 27). Moreover, the resulting outer loop accuracy was 82.8 ± 3.4% (accuracy of each fold: 80.6%, 80.9%, and 86.6%), which was higher than that using SVM with relative powers under the same nested cross-validation (mean accuracy, 78.8 ± 0.7%; each fold, 78.4%, 79.6%, and 78.5%; see Supplementary Table S5 and Fig. S9 for the confusion matrix and ROC curve for MNet classification, respectively).

## Discussion

We trained a novel deep neural network, MNet, to classify two neurological diseases and healthy subjects using big data from MEG signals. The trained MNet succeeded in classifying the neurological diseases with a high degree of accuracy and specificity. This is the first study to classify different neurological diseases according to one classifier using MEG signals. The high specificity for all diseases demonstrated that the MNet would be useful to improve the diagnosis of neurological diseases.

The MNet successfully classified neurological diseases with higher accuracy than the SVM. Although both classifiers used the same band powers as inputs, we suggest that the MNet extracted additional features in the convolution layer, which improved its classification accuracy. The network applies a large kernel that covers all of the channels at the beginning. This process may extract relationships within all of the channels, which we call global features, and the MNet’s successful classification suggests that it succeeded in extracting global features that characterize the diseases. In the previous studies, it has been demonstrated that a convolutional neural network is effective for time series data^37^ and achieves better accuracy in classifying wave forms, such as sound, than other methods using some conventional features^38,39^. The proposed convolutional neural network, MNet, will improve the classification of neurological diseases based on MEG signals and be useful for finding novel features to characterize them.

The classification accuracies evaluated in this study are comparable to those of previous studies. In a previous study using a Bonn university dataset^40^, EEG signals were classified between normal and interictal states with an accuracy of 97.3%^41^, which was slightly higher than that of our study. However, the Bonn university datasets were composed of only five healthy subjects and five patients with epilepsy, and the previous study used different segments of the same subjects’ data for training and testing. In contrast, our study included 67 healthy subjects, and 140 patients with epilepsy. Moreover, in our study the classification of patients with epilepsy and healthy subjects was performed by splitting subjects into training and testing datasets, so that neither dataset contained data from same subject. The MNet therefore not only classified the patients with epilepsy with comparable accuracy to the previous study, but also demonstrated a capability to generalize over patients.

It does not appear that our classification was dependent on either sex differences or age. The male-to-female ratios of patients with epilepsy and healthy subjects were both nearly one-to-one; and the classification accuracy between the patients with epilepsy and healthy subjects was 88.7 ± 9.3%. We also performed classification of patients with epilepsy and patients with SCI, both groups having a similar age distribution. The resulting classification accuracy was 79.8 ± 11.7%, which was also reasonably high. It appears, therefore, that our method can be applied regardless of age or sex.

It should be noted that our method was robust and transferable to different recording conditions. Indeed, we used five different recording conditions among three types of subjects. Even using the data recorded under different conditions, the MNet succeeded in classifying the diseases with high accuracy, indicating the robustness of our method. However, it might be difficult for the trained decoder to classify diseases using data recorded by another MEG scanner. Improvements to current source estimation and alignment techniques might make our method applicable for different MEG scanners^42^.

Deep learning from scratch is usually difficult with limited amounts of data. However, even with the limited amount of data in this study, we succeeded in classifying three types of subjects. One reason for this success was that we enlarged the data set by dividing the 220 s or 280 s data to 275 or 350 segments of 800-ms time data for each subject, allowing us to use about 65,000 data segments for training of the three classes, which was a comparable amount to MNIST^43^, the database of handwritten digits (0–9) often used for training deep neural networks, which suggests that we had a reasonable amount of data to train a network of this size.

However, the number of subjects might not be large enough to cover fluctuations such as the differences in patient symptoms or medicine dosage. Future work should therefore be performed with more subjects, because the performance quality of deep learning drastically improves with larger datasets^44^. In addition, our method might be improved with data from other modalities. A previous study suggested that MEG and EEG provide complementary information and it is ideal to use both^45^, while MRI or other modalities also provide additional useful information^46^. We will integrate data from different modalities in the future to improve the accuracy of automatic diagnoses. Moreover, other deep learning techniques such as transfer learning, generative models, data augmentation, and feature visualization could be used for future research to improve our system.

In conclusion, our method was effective for classifying healthy subjects and patients with two different neurological diseases. Using deep learning with big datasets including MEG signals will improve the diagnosis of various neurological diseases.

## Methods

### Participants

The study included 67 healthy subjects (35 women and 32 men, median age 60 years, range 21–86 years), 26 patients with SCI (3 women and 23 men, median age 34.5 years, range 22–61 years), and 140 patients with epilepsy (72 women and 68 men, median age 26.5 years, range 7–71 years; for age distribution, see Supplementary Fig. S10, and for detailed information of each record, see Supplementary Table S11). The subjects were recruited at the Osaka University hospital from April 2010 to October 2017. Diagnosis was performed by a specialist in neurology based on symptoms and neuroimaging. The criteria for defining healthy subjects were as follows: (1) no past history or symptoms of neurological diseases, (2) not having routinely prescribed medicine, and (3) having appropriate cognitive function according to the Japanese Adult Reading Test^47^. The study adhered to the Declaration of Helsinki and was performed in accordance with protocols approved by the Ethics Committee of Osaka University Clinical Trial Center (No. 14448, No. 15259, and No. 17441). All participants were informed of the purpose and possible consequences of this study, and written informed consent was obtained.

### MEG measurement

Each subject participated in multiple sessions to measure resting-state MEG signals in a day. During the sessions, the MEG signals were recorded with one of the following five measurement conditions: (1) sampling frequency of 2 k Hz with the low-pass filter at 500 Hz, the high-pass filter at 0.1 Hz, and the band-stop filter at 60 Hz; (2) sampling frequency of 1 k Hz with the low-pass filter at 200 Hz and the band-stop filter at 60 Hz; (3) sampling frequency of 1 k Hz with the low-pass filter at 200 Hz, the high-pass filter at 0.1 Hz, and the band-stop filter at 60 Hz; (4) sampling frequency of 2 k Hz with the low-pass filter at 500 Hz and the high-pass filter at 0.1 Hz; and (5) sampling frequency of 2 k Hz with the low-pass filter at 500 Hz and the high-pass filter at 0.1 Hz. Duration of the recording was either 240 s or 300 s. For any single subject, the same measurement condition and same duration were used throughout the sessions.

Measurements were performed by a 160-channel whole-head MEG equipped with coaxial-type gradiometers housed in a magnetically shielded room (MEGvision NEO; Yokogawa Electric Corporation, Kanazawa, Japan). The MEG channel positions are shown in Fig. 1. Five head marker coils were attached to the subject’s face before beginning the MEG measurement to provide the position and orientation of MEG sensors relative to the head. The positions of the five marker coils were measured to evaluate the differences in the head position before and after each session. The maximum acceptable difference was 5 mm.

During MEG measurements, subjects were in a supine position with the head centred in the MEG gantry. They were instructed to close their eyes, not to move their head. For the patients with SCI and healthy subjects, we instructed them not to think of anything in particular and not to fall asleep during the measurement. On the other hand, for patients with epilepsy, we instructed them to relax without thinking of anything in particular and allowed them to sleep. We simultaneously measured EEG of the epilepsy patients to monitor their sleep status.

### Data pre-processing

For each subject, we only used MEG signals recorded in one session (either 240 s or 300 s) in which the subject was awake. We applied the high-pass filter at 1 Hz and the low-pass filter at 50 Hz on the MEG signals so that filtered signals of all subjects contain the same frequency components among five different measurement conditions. We used the pop_eegfiltnew function in EEGLAB for filtering^48^. Moreover, sampling rates for all data were adjusted to 1 k Hz by down sampling. We discarded the first 10 s and the last 10 s of signals in order to avoid filter edge effect. Data were pre-processed by MATLAB R2015b (MathWorks, Natick, MA, USA).

### Network architecture

We developed the MNet based on previously reported model EnvNet-v2^38,39^, which is a convolutional neural network for classifying environmental sounds. The brief architecture of the MNet is shown in Fig. 1, and the detailed configuration of the MNet is shown in Table 4. The MNet extracted global features over all channels within the initial convolution layer, and some band powers from each channel were concatenated at the fully connected layer 13.

**Table 4:** Detailed configuration of MNet.

Layer	Ksize	Stride	# of filters	Data shape
Input				(1, 160, 800)
Conv1	(160, 64)	(1, 2)	32	(32, 1, 369)
Conv2	(1, 16)	(1, 2)	64	(64, 1, 177)
Pool2	(1, 2)	(1, 2)		(64, 1, 89)
Swap axes				(1, 64, 89)
Conv3	(8, 8)	(1, 1)	32	(32, 57, 82)
Conv4	(8, 8)	(1, 1)	32	(32, 50, 75)
Pool4	(5, 3)	(5, 3)		(32, 10, 25)
Conv5	(1, 4)	(1, 1)	64	(64, 10, 22)
Conv6	(1, 4)	(1, 1)	64	(64, 10, 19)
Pool6	(1, 2)	(1, 2)		(64, 10, 10)
Conv7	(1, 2)	(1, 1)	128	(128, 10, 9)
Conv8	(1, 2)	(1, 1)	128	(128, 10, 8)
Pool8	(1, 2)	(1, 2)		(128, 10, 4)
Conv9	(1, 2)	(1, 1)	256	(256, 10, 3)
Conv10	(1, 2)	(1, 1)	256	(256, 10, 2)
Pool10	(1, 2)	(1, 2)		(256, 10, 1)
Fc11	—	—	1,024	(1,024)
Fc12	—	—	1,024	(1,024)
Input				(1, 160, 800)
RPS				(1, 160, 6)
Concat				(1,984)*
Fc13	—	—	# of classes	(# of classes)

Ksize: kernel size; #: number; Conv: convolution; Pool: max pooling; Fc: fully connected; RPS: Relative power spectrum; Concat: concatenated.

^*^Concatenation of the output of Fc12 and RPS.

Input data for the MNet were 800-ms MEG signals consisted of 160 channels. Input data were processed in two ways: one by neural network and the other by Fourier transformation. In neural network processing, we extracted global features from the data by applying two spatial and temporal convolutional layers. We then treated the data like an image in time and frequency domains by swapping axes^38,39^, and applied eight more convolutional layers and then fully connected layers 11 and 12. For the Fourier transformation processing, input data was applied with fast Fourier transformation by CuPy^49^ to acquire powers in six frequency bands (δ: 1–4 Hz; θ: 4–8 Hz; low-α: 8–10 Hz; high-α: 10–13 Hz; β: 13–30 Hz; low-γ: 30–50 Hz) for each channel. The six powers were divided by the summation of the powers to yield the relative power for each channel, resulting in 960 decoding features (160 channels for each of the 6 frequency bands). The two forms of processed data were concatenated, before being thrown into the fully connected layer 13. Finally, we applied the softmax function, getting the probability of each disease. ReLU was applied to each layer.

Hyperparameters, MEG segment size, and max epoch were chosen based on our preliminary ECoG study, which classified the category of visual stimulus from ECoG signals using EnvNet, the original network for MNet. In that study, we compared the classification accuracy among seven ECoG signal segment sizes: 700, 750, 800, 850, 900, 950, and 1000 ms. Of these seven segment sizes, the classification accuracy was highest for 800-ms segment size, so we used 800-ms segment to classify the MEG signals. The dropout value (0.5) was the Chainer v5.00 default value^50^. For weight decay, we used the same value as the default settings of the EnvNet-v2^38,39^.

### Model training and testing

The performance of the MNet was evaluated by stratified 10-fold cross-validation^51^, by splitting patients into subjects for training and subjects for testing. In each training epoch of the MNet, 64 segments of 800-ms MEG signals were randomly extracted as input to the MNet from each subject for training. Each 800-ms segment was normalized to have a mean of zero and standard deviation one for each channel by the scikit-learn pre-processing function^52^. Because the number of subjects for each disease label was different, we balanced the numbers of segments among labels by simply using the same segments multiple times, in order to avoid a bias in the training dataset. Using these segments as input, we trained the MNet with the cross entropy criteria and a mini-batch algorithm^53^ with size 64. Momentum SGD with a momentum of 0.9 and learning rate of 0.001 was used as an optimizer. To avoid overfitting, we applied weight decay^54^ of 0.0005, batch normalization^55^ after fully connecting layers 11 and 12, and dropout^56^ of 50% before fully connecting layers 12 and 13. We initialized the weights of the MNet randomly. Training was terminated after 27 epochs.

To classify the disease label of each test subject with the trained MNet, we split whole MEG signals into segments using non-overlapping 800-ms time-windows. Each 800-ms segment was normalized to have a mean of zero and standard deviation of one in the same manner as in the normalization for the training data. Disease labels were predicted for each segment using the trained MNet. The predicted probabilities of diseases were averaged over all segments for each subject, resulting in one disease prediction for each single subject.

### Nested cross-validation

To confirm the validity of our method, nested cross-validation was performed for classifying patients with epilepsy and healthy subjects. The outer loop was three-fold, and the inner loop was two-fold. In the inner loops, the best weight decay among 0.005, 0.0005, and 0.00005 was chosen, and the best epoch was chosen within 30 epochs. To reduce the risk of choosing an extraordinary value, validation accuracy was averaged over the inner loops when choosing the best hyperparameters. In the outer loop, the model was re-trained using the training datasets with the best hyperparameters, and then the trained model tested the test dataset, which was separated.

### Decode from relative power using SVM

We classified disease labels using relative powers of MEG signals for each channel as decoding features, to compare with the accuracy achieved by the MNet. For each 800-ms segments used in the MNet testing, the MEG signals were applied with a Hamming window and fast Fourier transformation to acquire powers in six frequency bands (δ: 1–4 Hz; θ: 4–8 Hz; low-α: 8–10 Hz; high-α: 10–13 Hz; β: 13–30 Hz; low-γ: 30–50 Hz). Finally, for each time window and channel, the six powers were divided by the summation of the powers to be the relative power, resulting in 960 decoding features (160 channels by 6 frequency bands) for each time window. To classify the disease label of each patient from the power features, we used L2-regularized L2-loss SVM implemented in Liblinear^57^, and 10-fold nested cross-validation. The split of the subjects in the outer cross-validation were kept the same to the split in the MNet testing for the comparison of classification accuracies. The SVM model was trained using decoding features from all segments within the training dataset. The penalty term of the SVM model was optimized using inner cross-validation, so that the penalty term was selected independently from the testing dataset in the outer cross-validation. To predict the disease label for each patient, decoding features from all 800-ms segments were classified, and majority voting was performed to determine one disease label. Finally, the classification accuracy was compared to that of the MNet using single-sided Wilcoxon signed-rank test.

### Code availability

The code used in this study is available by contacting the corresponding author (T.Y.).

## Supplementary information

Supplementary information

## Data Availability

The data that support the findings of this study are available on request from the corresponding author (T.Y.). The data are not publicly available because they contain information that could compromise the research participants’ privacy and/or consent.
