# Supplementary material for: Automatic diagnosis of neurological diseases using MEG signals with a deep neural network
Source: Sci Rep. 2019 Mar 25;9:5057. doi: 10.1038/s41598-019-41500-x (PMC6433906; doi:10.1038/s41598-019-41500-x)
Supplement: Supplementary file 1 — Supplementary information [file 41598_2019_41500_MOESM1_ESM.pdf]

Supplementary Information

Automatic diagnosis of neurological diseases using MEG signals with a deep neural network

Jo Aoe[1†], Ryohei Fukuma[2†], Takufumi Yanagisawa[1,2,3]*, Tatsuya Harada[4]*, Masataka Tanaka[2], Maki Kobayashi[2], You Inoue[2], Shota Yamamoto[2], Yuichiro Ohnishi[2], Haruhiko Kishima[2]

[1]Osaka University Institute for Advanced Co-Creation Studies, Suita, Japan

[2]Department of Neurosurgery, Osaka University Graduate School of Medicine, Suita, Japan

[3]JST PRESTO, Suita, Japan

[4]Department of Mechano-Informatics, Graduate School of Information Science and Technology, The University of Tokyo

[†] These authors contributed equally to this work.

*** Correspondence:**

Takufumi Yanagisawa, M.D., Ph.D., tyanagisawa@nsurg.med.osaka-u.ac.jp

Tatsuya Harada, Ph.D., harada@mi.t.u-tokyo.ac.jp

Supplementary Table S1.

Confusion matrix of the classification of healthy subjects, patients with epilepsy, and patients with spinal cord injury.

		Predicted		
		HS	EP	SCI
Original	HS	53	10	4
	EP	9	123	8
	SCI	11	3	12

HS: healthy subjects; EP: patients with epilepsy; SCI: patients with spinal cord injury

Supplementary Table S2.

Confusion matrix of the classification of healthy subjects and patients with epilepsy.

		Predicted	
		HS	EP
Original	HS	58	9
	EP	13	127

HS: healthy subjects; EP: patients with epilepsy

Supplementary Table S3.
Confusion matrix of the classification of healthy subjects and patients with spinal cord injury.

		Predicted	
		HS	SCI
Original	HS	55	12
	SCI	16	10

HS: healthy subjects; SCI: patients with spinal cord injury

Supplementary Table S4.

Confusion matrix of the classification of patients with epilepsy and patients with spinal cord injury.

		Predicted	
		EP	SCI
Original	EP	137	3
	SCI	10	16

EP: Patients with epilepsy; SCI: patients with spinal cord injury

Supplementary Table S5.

Confusion matrix of the classification of healthy subjects and patients with epilepsy by three-fold nested cross-validation.

		Predicted	
		HS	EP
Original	HS	50	17
	EP	13	127

HS: healthy subjects; EP: patients with epilepsy

Supplementary Figure S6.

ROC curve of the classification of healthy subjects and patients with epilepsy; in this figure, positive means having epilepsy

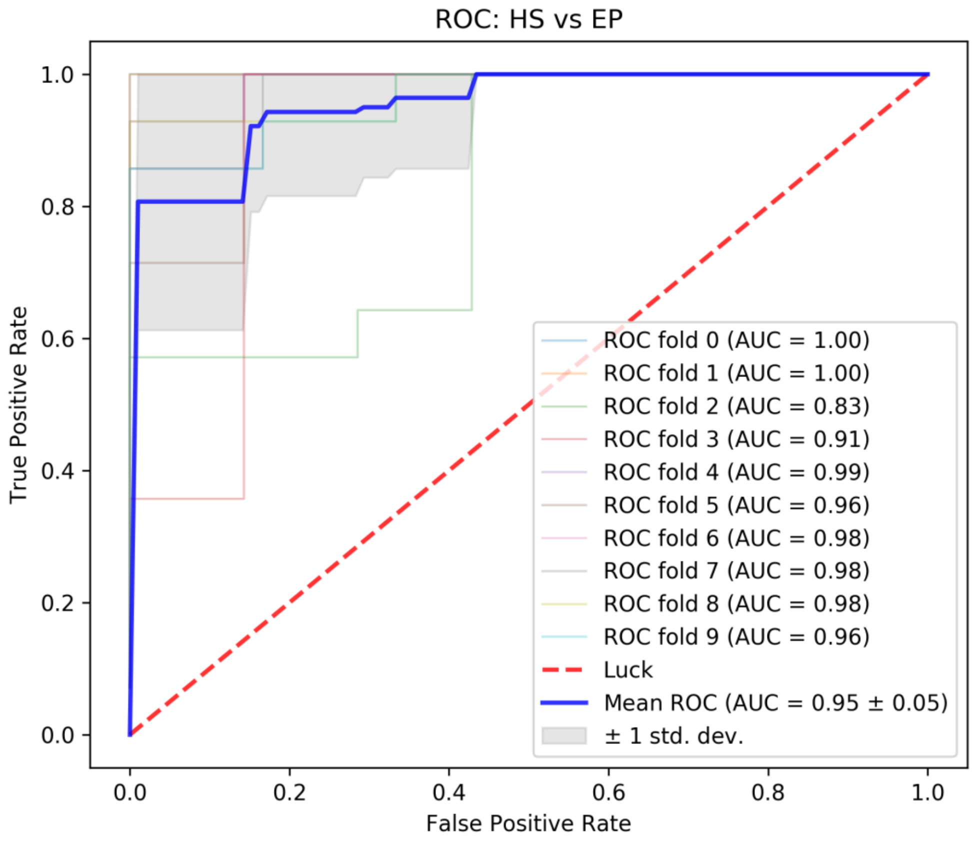

AUC: area under the curve; HS: healthy subjects; EP: patients with epilepsy

Supplementary Figure S7.

ROC curve of the classification of healthy subjects and patients with spinal cord injury; in this figure, positive means having spinal cord injury.

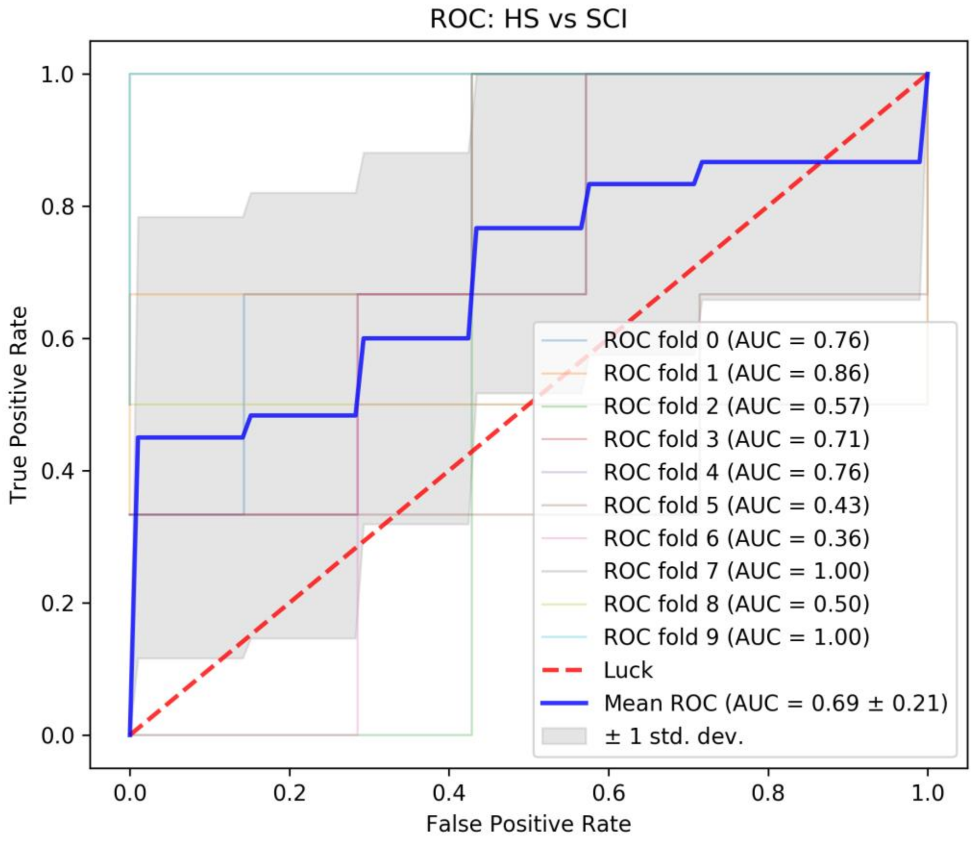

AUC: area under the curve; HS: healthy subjects; SCI: patients with spinal cord injury

Supplementary Figure S8.

ROC curve of the classification of patients with epilepsy and patients with spinal cord injury; in this figure, positive means having spinal cord injury.

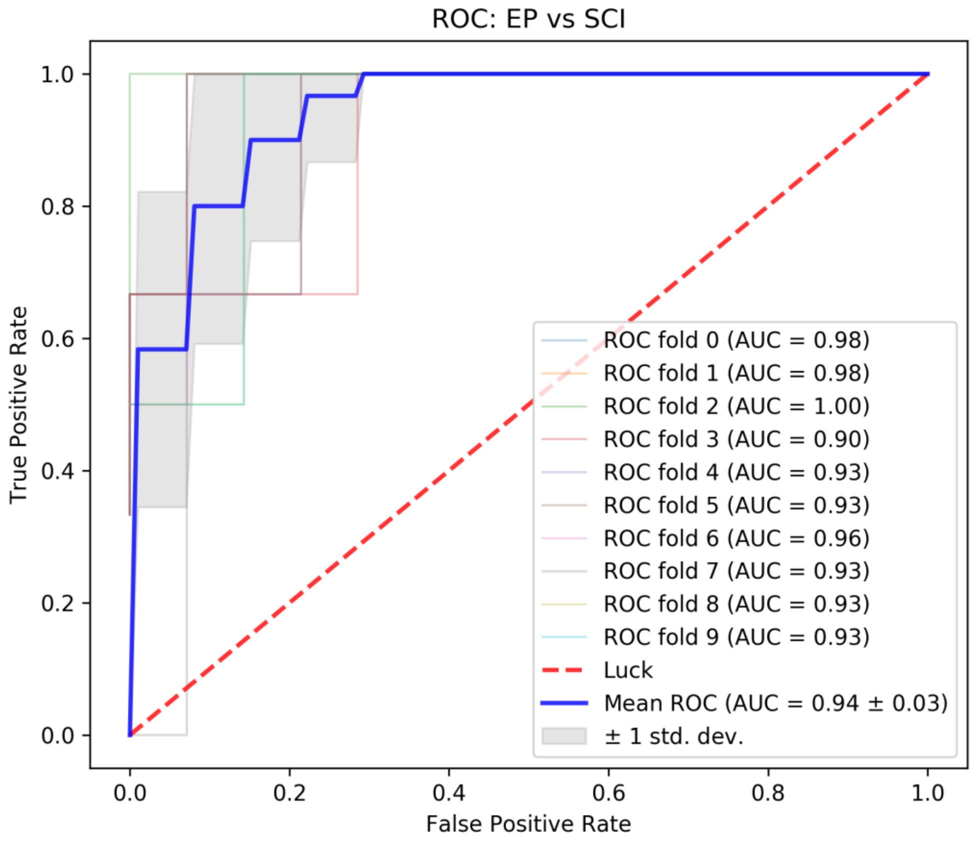

AUC: area under the curve; EP: patients with epilepsy; SCI: patients with spinal cord injury

Supplementary Figure S9.

ROC curve of the classification of patients with epilepsy and healthy subjects with three-fold nested cross-validation; in this figure, positive means having epilepsy.

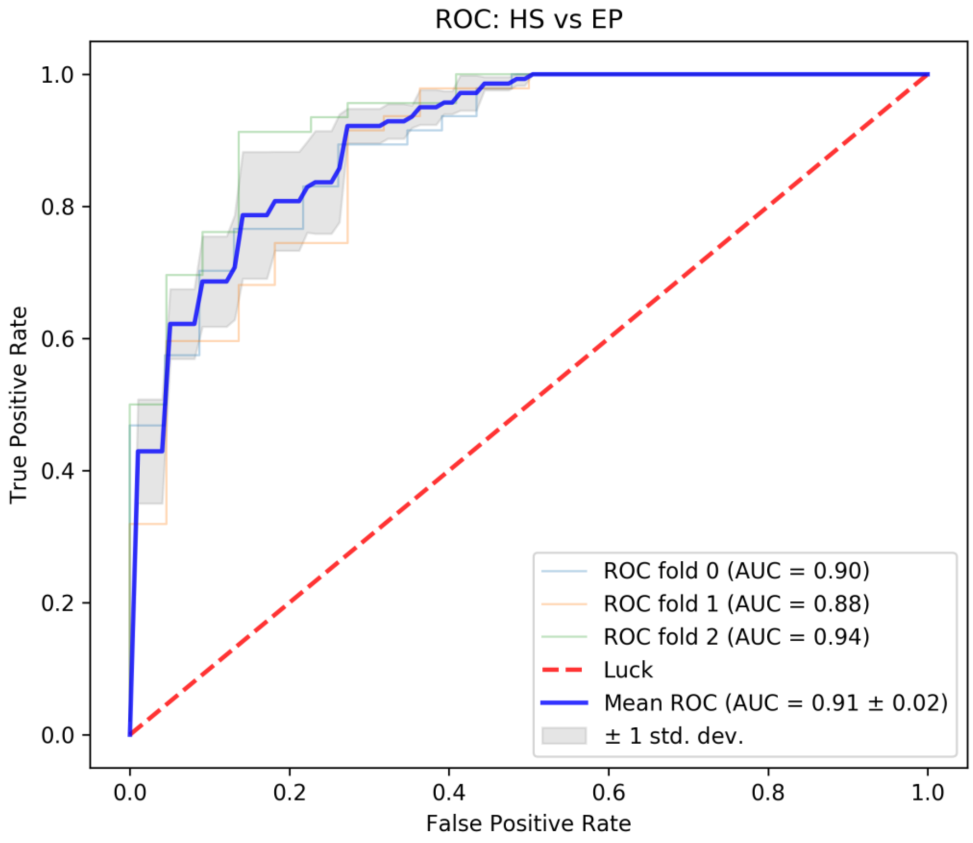

AUC: area under the curve; EP: patients with epilepsy; HS: healthy subjects

Supplementary Figure S10. Box and whisker plots of age distribution of healthy subjects, patients with spinal cord injury, and patients with epilepsy. The box extends from the lower to upper quartile values of the data, with a line at the median. The whiskers extend from the box to show the range of the data. Outliers are represented as circles.

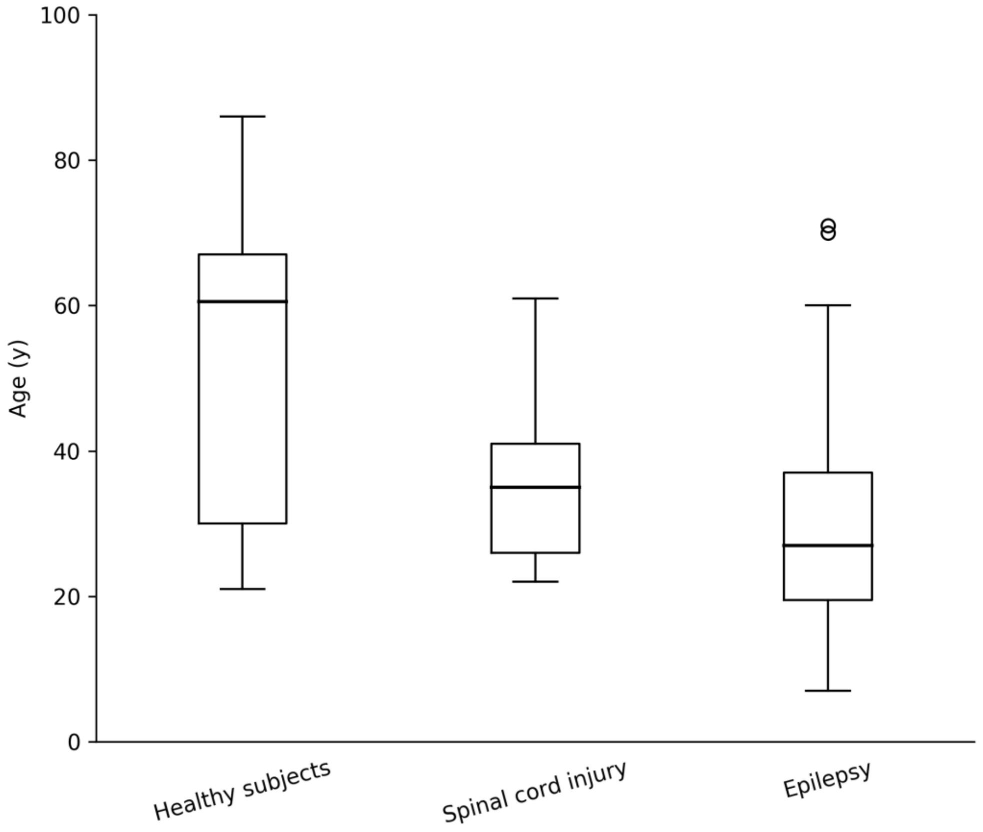

Supplementary Table S11. Records information of data used in the study. Subject ID numbers subjects individually. The high-pass and low-pass filter columns show the cut-off frequencies of the high- and low-pass filters, respectively, and the band elimination filter column shows the frequency of the band elimination filter.

Subject ID	Diagnosis	Age (y)	Gender	Sampling Rate (Hz)	Acquisition Time (s)	High pass filter (Hz)	Low pass filter (Hz)	elimination filter	Subtypes of epilepsy
1	Healthy subject	67	Male	2k	300	0.1	500	N/A	
2	Healthy subject	62	Female	2k	300	0.1	500	N/A	
3	Healthy subject	72	Male	2k	300	0.1	500	N/A	
4	Healthy subject	66	Female	2k	300	0.1	500	N/A	
5	Healthy subject	67	Male	2k	300	0.1	500	N/A	
6	Healthy subject	68	Male	2k	300	0.1	500	N/A	
7	Healthy subject	66	Male	2k	300	0.1	500	N/A	
8	Healthy subject	69	Male	2k	300	0.1	500	N/A	
9	Healthy subject	60	Female	2k	300	0.1	500	N/A	
10	Healthy subject	65	Male	2k	300	0.1	500	N/A	
11	Healthy subject	70	Male	2k	300	0.1	500	N/A	
12	Healthy subject	55	Male	2k	300	0.1	500	N/A	
13	Healthy subject	56	Female	2k	300	0.1	500	N/A	
14	Healthy subject	69	Female	2k	300	0.1	500	N/A	
15	Healthy subject	72	Male	2k	300	0.1	500	N/A	
16	Healthy subject	67	Male	2k	300	0.1	500	N/A	
17	Healthy subject	69	Male	2k	300	0.1	500	N/A	
18	Healthy subject	57	Female	2k	300	0.1	500	N/A	
19	Healthy subject	64	Female	2k	300	0.1	500	N/A	
20	Healthy subject	74	Female	2k	240	0.1	500	N/A	
21	Healthy subject	76	Male	2k	240	0.1	500	N/A	
22	Healthy subject	71	Male	2k	240	0.1	500	N/A	
23	Healthy subject	55	Female	2k	240	0.1	500	N/A	
24	Healthy subject	63	Female	2k	240	0.1	500	N/A	

25	Healthy subject	57	Female	2k	240	0.1	500	N/A	
26	Healthy subject	57	Female	2k	240	0.1	500	N/A	
27	Healthy subject	30	Male	2k	240	0.1	500	N/A	
28	Healthy subject	33	Male	1k	300	N/A	200	60	
29	Healthy subject	34	Female	1k	300	N/A	200	60	
30	Healthy subject	65	Male	2k	240	0.1	500	N/A	
31	Healthy subject	63	Female	2k	240	0.1	500	60	
32	Healthy subject	64	Female	2k	240	0.1	500	60	
33	Healthy subject	61	Male	2k	240	0.1	500	60	
34	Healthy subject	63	Male	2k	240	0.1	500	60	
35	Healthy subject	65	Female	2k	240	0.1	500	60	
36	Healthy subject	62	Female	2k	240	0.1	500	60	
37	Healthy subject	59	Male	2k	240	0.1	500	60	
38	Healthy subject	66	Male	2k	240	0.1	500	60	
39	Healthy subject	56	Male	2k	240	0.1	500	60	
40	Healthy subject	66	Male	2k	240	0.1	500	60	
41	Healthy subject	59	Male	2k	240	0.1	500	60	
42	Healthy subject	69	Male	2k	240	0.1	500	60	
43	Healthy subject	68	Female	2k	240	0.1	500	60	
44	Healthy subject	84	Female	2k	240	0.1	500	60	
45	Healthy subject	40	Male	2k	240	0.1	500	60	
46	Healthy subject	58	Female	2k	240	0.1	500	60	
47	Healthy subject	86	Male	2k	240	0.1	500	60	
48	Healthy subject	80	Male	2k	240	0.1	500	60	
49	Healthy subject	21	Female	1k	300	0.1	200	60	
50	Healthy subject	21	Female	2k	240	0.1	500	60	
51	Healthy subject	23	Female	2k	240	0.1	500	60	
52	Healthy subject	22	Female	1k	300	N/A	200	60	
53	Healthy subject	24	Female	2k	240	0.1	500	60	
54	Healthy subject	23	Male	2k	240	0.1	500	60	
55	Healthy subject	22	Female	2k	240	0.1	500	60	
56	Healthy subject	22	Female	2k	240	0.1	500	60	

57	Healthy subject	21	Female	2k	240	0.1	500	60	
58	Healthy subject	29	Male	1k	300	N/A	200	60	
59	Healthy subject	22	Female	1k	300	N/A	200	60	
60	Healthy subject	25	Female	1k	300	N/A	200	60	
61	Healthy subject	24	Female	1k	300	N/A	200	60	
62	Healthy subject	37	Male	1k	300	N/A	200	60	
63	Healthy subject	30	Male	1k	300	N/A	200	60	
64	Healthy subject	25	Female	1k	300	N/A	200	60	
65	Healthy subject	22	Female	1k	300	N/A	200	60	
66	Healthy subject	22	Female	1k	300	N/A	200	60	
67	Healthy subject	22	Female	1k	300	N/A	200	60	
68	Spinal cord injury	38	Male	1k	300	N/A	200	60	
69	Spinal cord injury	28	Male	1k	300	N/A	200	60	
70	Spinal cord injury	35	Male	1k	300	N/A	200	60	
71	Spinal cord injury	33	Male	1k	300	N/A	200	60	
72	Spinal cord injury	25	Male	1k	300	N/A	200	60	
73	Spinal cord injury	41	Female	1k	300	N/A	200	60	
74	Spinal cord injury	22	Male	2k	240	0.1	500	60	
75	Spinal cord injury	61	Male	2k	240	0.1	500	60	
76	Spinal cord injury	61	Male	2k	240	0.1	500	60	
77	Spinal cord injury	23	Male	2k	240	0.1	500	60	
78	Spinal cord injury	22	Male	2k	240	0.1	500	60	
79	Spinal cord injury	48	Male	2k	240	0.1	500	60	
80	Spinal cord injury	32	Male	1k	300	N/A	200	60	
81	Spinal cord injury	46	Male	2k	240	0.1	500	60	
82	Spinal cord injury	43	Male	2k	240	0.1	500	60	
83	Spinal cord injury	23	Female	2k	240	0.1	500	60	
84	Spinal cord injury	34	Male	2k	240	0.1	500	60	
85	Spinal cord injury	40	Male	1k	300	N/A	200	60	
86	Spinal cord injury	22	Male	1k	300	N/A	200	60	
87	Spinal cord injury	36	Male	1k	300	N/A	200	60	
88	Spinal cord injury	39	Female	1k	300	N/A	200	60	

89	Spinal cord injury	39	Male	1k	240	0.1	200	60	
90	Spinal cord injury	45	Male	1k	240	0.1	200	60	
91	Spinal cord injury	29	Male	1k	300	N/A	200	60	
92	Spinal cord injury	26	Male	1k	300	N/A	200	60	
93	Spinal cord injury	22	Male	2k	240	0.1	500	60	
94	Epilepsy	27	Male	2k	240	0.1	500	60	Right TLE
95	Epilepsy	18	Female	1k	240	0.1	200	60	TLE
96	Epilepsy	49	Male	2k	240	0.1	500	60	Rt TLE
97	Epilepsy	59	Male	2k	240	0.1	500	60	Lt Frontal Epi
98	Epilepsy	30	Female	1k	240	0.1	200	60	Lt TLE
99	Epilepsy	22	Female	2k	240	0.1	500	60	Lt TLE susp
100	Epilepsy	34	Female	2k	240	0.1	500	60	Lt TLE
101	Epilepsy	17	Male	2k	240	0.1	500	60	JME
102	Epilepsy	45	Male	2k	240	0.1	500	60	Left SMA Epi
103	Epilepsy	19	Female	2k	240	0.1	500	60	Frontal epilepsy
104	Epilepsy	21	Male	2k	240	0.1	500	60	bil. TLE
105	Epilepsy	19	Male	2k	240	0.1	500	60	Rt TLE
106	Epilepsy	32	Female	2k	240	0.1	500	60	Rt motor FCD
107	Epilepsy	21	Male	2k	240	0.1	500	60	Rt TLE
108	Epilepsy	26	Male	2k	240	0.1	500	60	Rt MTLE
109	Epilepsy	58	Male	2k	240	0.1	500	60	Lt Occipital
110	Epilepsy	40	Female	2k	240	0.1	500	60	Rt TLE
111	Epilepsy	33	Female	2k	240	0.1	500	60	bil TLE
112	Epilepsy	25	Male	2k	240	0.1	500	60	Intractable Epi
113	Epilepsy	17	Male	2k	240	0.1	500	60	Rt Frontal
114	Epilepsy	20	Male	2k	240	0.1	500	60	Lt TLE
115	Epilepsy	21	Female	2k	240	0.1	500	60	Idiopathic epilepsy
116	Epilepsy	21	Male	2k	240	0.1	500	60	Lt TLE susp
117	Epilepsy	17	Female	2k	240	0.1	500	60	Lt Front FCD
118	Epilepsy	26	Female	2k	240	0.1	500	60	Lt Occipital
119	Epilepsy	20	Female	2k	240	0.1	500	60	Rt Occip FCD
120	Epilepsy	16	Male	2k	240	0.1	500	60	Rt Occip Tumor

121	Epilepsy	56	Female	2k	240	0.1	500	60	Trauma Rt Par
122	Epilepsy	52	Female	2k	240	0.1	500	60	LT TLE
123	Epilepsy	23	Female	2k	240	0.1	500	60	JME
124	Epilepsy	28	Male	2k	240	0.1	500	60	Rt Frontal FCD
125	Epilepsy	33	Female	2k	240	0.1	500	60	Symptomatic localization related epilepsy
126	Epilepsy	24	Male	2k	240	0.1	500	60	Rt TLE
127	Epilepsy	21	Male	2k	240	0.1	500	60	Rt Temp DNT
128	Epilepsy	27	Male	2k	240	0.1	500	60	Idiopathic epilepsy
129	Epilepsy	19	Male	2k	240	0.1	500	60	Lt TLE
130	Epilepsy	60	Female	2k	240	0.1	500	60	Rt TLE susp
131	Epilepsy	43	Female	2k	240	0.1	500	60	Lt TLE susp
132	Epilepsy	7	Male	2k	240	0.1	500	60	Lt TLE
133	Epilepsy	50	Female	2k	240	0.1	500	60	Lt TLE
134	Epilepsy	22	Male	2k	240	0.1	500	60	Rt TLE
135	Epilepsy	30	Male	2k	240	0.1	500	60	Rt Frontal
136	Epilepsy	45	Male	2k	240	0.1	500	60	bil Parietal FCD
137	Epilepsy	22	Male	2k	240	0.1	500	60	Frontal lobe epilepsy after encephalitis
138	Epilepsy	29	Female	2k	240	0.1	500	60	Idiopathic generalized epilepsy
139	Epilepsy	22	Male	2k	240	0.1	500	60	Rt Front Parietal
140	Epilepsy	29	Female	2k	240	0.1	500	60	Idiopathic generalized epilepsy
141	Epilepsy	53	Female	2k	240	0.1	500	60	Trauma TLE
142	Epilepsy	12	Male	2k	240	0.1	500	60	Lt TLE
143	Epilepsy	35	Female	2k	240	0.1	500	60	after right old infarction
144	Epilepsy	13	Female	2k	240	0.1	500	60	Lt TLE
145	Epilepsy	21	Female	2k	240	0.1	500	60	Rt TLE susp
146	Epilepsy	37	Male	2k	240	0.1	500	60	Lt TLE
147	Epilepsy	40	Female	2k	240	0.1	500	60	Bil TLE
148	Epilepsy	22	Male	2k	240	0.1	500	60	Rt TLE

149	Epilepsy	20	Female	2k	240	0.1	500	60	Lt TLE susp
150	Epilepsy	15	Female	2k	240	0.1	500	60	Lt TLE susp
151	Epilepsy	21	Male	2k	240	0.1	500	60	epilepsy after encephalitis
152	Epilepsy	35	Male	2k	240	0.1	500	60	Lt TLE susp
153	Epilepsy	43	Male	2k	240	0.1	500	60	Related to Rt localization
154	Epilepsy	32	Female	2k	240	0.1	500	60	epilepsy after encephalitis
155	Epilepsy	35	Female	2k	240	0.1	500	60	Bil TLE
156	Epilepsy	52	Female	2k	240	0.1	500	60	Lt TLE
157	Epilepsy	25	Male	2k	240	0.1	500	60	Lt PCC-Hip FCD
158	Epilepsy	23	Female	2k	240	0.1	500	60	Bil Front
159	Epilepsy	43	Male	2k	240	0.1	500	60	Left Medial TLE
160	Epilepsy	32	Male	2k	240	0.1	500	60	Left Lateral TLE
161	Epilepsy	31	Female	2k	240	0.1	500	60	Medial+Lateral Right TLE
162	Epilepsy	19	Male	1k	240	0.1	200	60	Left Lateral TLE
163	Epilepsy	43	Female	2k	240	0.1	500	60	Right Medial TLE
164	Epilepsy	45	Female	1k	300	N/A	200	60	Left Medial TLE
165	Epilepsy	12	Male	1k	300	N/A	200	60	Left Medial TLE
166	Epilepsy	17	Male	1k	240	0.1	200	60	Left Medial TLE
167	Epilepsy	37	Male	1k	240	0.1	200	60	Left Medial TLE
168	Epilepsy	34	Female	1k	240	0.1	200	60	Left Medial TLE
169	Epilepsy	26	Female	1k	240	0.1	200	60	Left Medial TLE
170	Epilepsy	39	Female	2k	240	0.1	500	60	Left Medial TLE
171	Epilepsy	39	Male	1k	240	0.1	200	60	Left Medial TLE
172	Epilepsy	21	Male	2k	240	0.1	500	60	Left Medial TLE
173	Epilepsy	24	Male	2k	240	0.1	500	60	Left Medial TLE
174	Epilepsy	30	Female	2k	240	0.1	500	60	Left Medial TLE
175	Epilepsy	22	Female	1k	300	N/A	200	60	Right Medial TLE
176	Epilepsy	33	Female	1k	300	N/A	200	60	Right Medial TLE
177	Epilepsy	19	Female	1k	240	0.1	200	60	Right Medial TLE
178	Epilepsy	19	Female	1k	240	0.1	200	60	Right Medial TLE
179	Epilepsy	30	Male	1k	240	0.1	200	60	Right Medial TLE
180	Epilepsy	13	Female	1k	240	0.1	200	60	Right Medial TLE

181	Epilepsy	21	Male	1k	240	0.1	200	60	Right Medial TLE
182	Epilepsy	50	Female	1k	240	0.1	200	60	Right Medial TLE
183	Epilepsy	21	Male	1k	240	0.1	200	60	Right Medial TLE
184	Epilepsy	70	Female	1k	240	0.1	200	60	Right Medial TLE
185	Epilepsy	42	Female	2k	240	0.1	500	60	Right Medial TLE
186	Epilepsy	40	Female	2k	240	0.1	500	60	Right Medial TLE
187	Epilepsy	15	Female	2k	240	0.1	500	60	Right Medial TLE
188	Epilepsy	19	Male	2k	240	0.1	500	60	Right Medial TLE
189	Epilepsy	35	Female	2k	240	0.1	500	60	Right Medial TLE
190	Epilepsy	12	Female	2k	240	0.1	500	60	Right Medial TLE
191	Epilepsy	40	Female	2k	240	0.1	500	60	Right Medial TLE
192	Epilepsy	20	Female	1k	240	0.1	200	60	Bilateral Medial TLE
193	Epilepsy	16	Female	1k	240	0.1	200	60	Right Lateral TLE
194	Epilepsy	37	Male	1k	240	0.1	200	60	Right Lateral TLE
195	Epilepsy	29	Female	1k	300	N/A	200	60	Left Lateral TLE
196	Epilepsy	15	Male	1k	240	0.1	200	60	Left Lateral TLE
197	Epilepsy	40	Male	2k	240	0.1	500	60	Left Lateral TLE
198	Epilepsy	35	Male	2k	240	0.1	500	60	Left Lateral TLE
199	Epilepsy	56	Male	1k	240	0.1	200	60	Bilateral Lateral TLE
200	Epilepsy	13	Female	1k	240	0.1	200	60	Medial+Lateral Left TLE
201	Epilepsy	27	Female	1k	240	0.1	200	60	Medial+Lateral Right TLE
202	Epilepsy	31	Female	1k	240	0.1	200	60	Medial+Lateral Right TLE
203	Epilepsy	35	Female	1k	240	0.1	200	60	Medial+Lateral Right TLE
204	Epilepsy	27	Female	1k	240	0.1	200	60	Medial+Lateral Right TLE
205	Epilepsy	45	Male	1k	240	0.1	200	60	Medial+Lateral Right TLE
206	Epilepsy	36	Male	2k	240	0.1	500	60	Medial+Lateral Right TLE
207	Epilepsy	71	Female	1k	240	0.1	200	60	non-TLE epilepsy
208	Epilepsy	14	Female	2k	240	0.1	500	60	non-TLE epilepsy
209	Epilepsy	34	Male	2k	240	0.1	500	60	unclassifiable Left
210	Epilepsy	36	Male	2k	240	0.1	500	60	unclassifiable Left
211	Epilepsy	22	Male	2k	240	0.1	500	60	unclassifiable Left
212	Epilepsy	19	Male	2k	240	0.1	500	60	unclassifiable Left

213	Epilepsy	38	Male	2k	240	0.1	500	60	unclassifiable Left
214	Epilepsy	17	Female	1k	240	0.1	200	60	unclassifiable Left
215	Epilepsy	18	Female	2k	240	0.1	500	60	unclassifiable Left
216	Epilepsy	16	Male	2k	240	0.1	500	60	unclassifiable Left
217	Epilepsy	46	Male	2k	240	0.1	500	60	unclassifiable Left
218	Epilepsy	15	Female	2k	240	0.1	500	60	unclassifiable Left
219	Epilepsy	52	Female	2k	240	0.1	500	60	unclassifiable Left
220	Epilepsy	57	Female	1k	240	0.1	200	60	unclassifiable Right
221	Epilepsy	34	Female	1k	240	0.1	200	60	unclassifiable Right
222	Epilepsy	15	Female	1k	240	0.1	200	60	unclassifiable Right
223	Epilepsy	46	Male	2k	240	0.1	500	60	unclassifiable Right
224	Epilepsy	23	Male	2k	240	0.1	500	60	unclassifiable Right
225	Epilepsy	11	Male	2k	240	0.1	500	60	unclassifiable Right
226	Epilepsy	25	Male	2k	240	0.1	500	60	unclassifiable Right
227	Epilepsy	22	Female	2k	240	0.1	500	60	unclassifiable Right
228	Epilepsy	14	Female	2k	240	0.1	500	60	unclassifiable Right
229	Epilepsy	20	Male	2k	240	0.1	500	60	unclassifiable Right
230	Epilepsy	27	Male	1k	240	0.1	200	60	unknown
231	Epilepsy	18	Female	1k	240	0.1	200	60	unknown
232	Epilepsy	16	Male	2k	240	0.1	500	60	unknown
233	Epilepsy	33	Male	2k	240	0.1	500	60	unknown
